# Barriers and facilitators to implementing an evidence-based woman-focused intervention in South African health services

**DOI:** 10.1186/s12913-017-2669-2

**Published:** 2017-11-21

**Authors:** Brittni N. Howard, Richard Van Dorn, Bronwyn J. Myers, William A. Zule, Felicia A. Browne, Tara Carney, Wendee M. Wechsberg

**Affiliations:** 1 Substance Use, Gender, and Applied Research Program, RTI International, 3040 E. Cornwallis Road, Research Triangle Park, NC 27709-2194 USA; 2 Behavioral and Urban Health Program, RTI International, 3040 E. Cornwallis Road, Research Triangle Park, NC 27709-2194 USA; 3 Alcohol, Tobacco and Other Drug Research Unit, South African Medical Research Council, Francie van Zijl Drive, Tygerberg, Cape Town 7505 South Africa

**Keywords:** HIV, Prevention intervention, Substance use, Women, Implementation science

## Abstract

**Background:**

Since the beginning of the HIV epidemic, numerous behavior change, risk-reduction, and biomedical interventions have been developed and tested. While some of these interventions have shown to be efficacious in randomized trials, it often takes almost two decades for an intervention to be translated into practice. Meanwhile, South Africa continues to have among the highest prevalence of HIV globally, with women of childbearing age bearing the burden of the epidemic. Given the urgency of the HIV epidemic among vulnerable women in South Africa, it is imperative that evidence-based interventions be implemented rapidly into practice. This study presents a first step toward examining the acceptability and feasibility of implementing the Women’s Health CoOp (WHC) in clinics and substance abuse rehab settings in Cape Town, South Africa.

**Methods:**

We conducted focus group discussions with women who use substances and with service providers, we also conducted in-depth interviews with health service planners. Our goal was to examine implementation and clinical outcomes associated with delivery of the WHC across clinics and substance abuse rehab programs.

**Results:**

All participants agreed on the need for the WHC. Perceived facilitators to implementing the WHC included the recognizable need for programs to empower women and to build the capacity of staff to address issues of substance use, sexual risk, and intimate partner violence. Participants also identified potential barriers to women engaging with this program, including the stigma women experience when seeking services and the lack of person-centered care at healthcare facilities.

**Conclusions:**

In a country with the largest number of women of childbearing age living with HIV, an evidence-based woman-focused intervention that comprehensively addresses women’s risk for suboptimal antiretroviral adherence may be essential for reducing HIV incidence. However, potential barriers to implementing the WHC successfully must be addressed before the program can be fully integrated into the services delivered by healthcare facilities.

**Trial registration:**

Clinical trials NCT02733003. Date of Registration: January 21, 2016, registered retroactively after participant enrollment.

## Background

More than 30 years has passed since the quest began for the discovery and cure for HIV. During these years, numerous behavior change, risk-reduction, and biomedical interventions have been developed and tested. Some have proven efficacious in randomized controlled trial and field settings; however, it often takes almost two decades for these evidence-based interventions to be translated into practice [1]. Despite these advances, South Africa continues to have among the highest prevalence of HIV globally [2] and a persistently high rate of HIV incident cases [3]. HIV is most commonly transmitted heterosexually in South Africa and it is concentrated disproportionately among women of childbearing age [3]. Women are more vulnerable than men because they are more susceptible anatomically and because gender inequality and intimate partner violence (IPV) are prevalent in South Africa; well-documented risk factors for HIV acquisition [4] that make it difficult for women to negotiate condom use with male sex partners [5].

Alcohol and other drug (AOD) use is also highly prevalent in South Africa. Estimates indicate that 13% of the South African adult population will meet diagnostic criteria for a substance use disorder in their lifetime [6], and the country has among the highest rates of hazardous and harmful alcohol use globally [7, 8]. Vulnerable women may cope with their marginal position in South African society by using AODs [5, 9], which contributes to HIV transmission [10, 11] by decreasing condom use and increasing risky sex behaviors [10]. For example, women may use AODs to cope with sex trading, which increases multiple risks for HIV exposure, or they may trade sex for AODs or for life necessities [12]. For women living with HIV, AOD use also has a negative impact on HIV treatment initiation and antiretroviral therapy (ART) adherence [13–15], and because AOD use is associated with risky sex behavior it increases the likelihood of onward transmission of HIV [16]. Consequently, there is a public health imperative to reduce AOD use and associated sex risk behavior among women living with HIV.

Several interventions have sought to address AOD use, risky sex behaviors and/or IPV among people living with or at risk for acquiring HIV in South Africa [17–20]. However, none of these interventions has targeted vulnerable women while addressing the nexus of these behaviors. A recent review argued that where AOD-using women face multiple and syndemic risks for HIV, including risky sex behaviors and IPV, evidence-based woman-focused interventions are needed to prevent new incident infections [21, 22]. The Women’s Health CoOp (WHC) is one such evidence-based woman-focused intervention.

### The Women’s Health CoOp

Based on the original Women’s CoOp, which was developed for use with African American women who use AODs [23], this two-session best-evidence empowerment-based intervention [24] has been adapted for use among vulnerable South African women [25–27]. The WHC is a cue-card delivered intervention that combines risk-reduction information for AODs, HIV and other sexually transmitted infections, and gender-based violence with behavioral skills training for sexual protection and safer sex communication. The adaptation for this study is being delivered in group format by a trained clinic interventionist. Each session lasts approximately 60 min, depending on the length of the discussion, role playing, and rehearsal of skills. See Table 1 for an overview of the WHC.

**Table 1:** Overview of the WHC

Session I		
• Why is reaching women so important?	• Concerns about HIV testing	ACTIVITY:
• Reasons women are at risk in South Africa	• Meaning of HIV test results	STI photos
• How do women get infected with HIV?	• South Africa has a very high rate of	ACTIVITY:
• What we want you to know about HIV	TB coinfection	Practice using male/female condoms
• Myths and truths about HIV/AIDS	• Preventing the spread of HIV	
• CD4 and viral load tests/ART	• Male and female condoms	ACTIVITY:
• Do you know what STIs look like?	• Oral protection for women	Roleplay sexual negotiation
• Keeping your private parts healthy	• Reducing sex risks	
• Circumcision decreases risk	• Negotiate for sexy safer sex	
Session II		
• Alcohol and drug abuse in South Africa	• Responses to conflict (fair fighting)	ACTIVITY:
• Alcohol and drug use compromises	• Concerns about abuse of women	Roleplay problem solving
behavior	• Myths and truths about abuse	
• Why is alcohol so risky for women?	• Rape and violence prevention	ACTIVITY:
• Alcohol and drugs affect unborn babies	• Sexual safety	Personalized Action Plan for women
• Facts about drug use and injection risk	• Problem-solving steps for life	
• Having a balanced life	• Standing your ground	
• Reducing alcohol and drug risks	• Stay alert, stay powerful	
• Harm reduction strategies	• Benefits of support: sister to sister	
• Benefits of rehab	• Women can become powerful	

Several randomized field trials have found the WHC to be efficacious for reducing AOD use and sex risk behavior among vulnerable women in South Africa [25–28], suggesting that the WHC could help prevent incident infections among vulnerable women if scaled up and implemented more widely.

Publicly funded primary healthcare (PHC) clinics that provide free HIV testing and counseling (HTC), antenatal care and free ART are often next door to substance abuse rehabilitation, or rehab, services. Consequently, using these services for women living with HIV is logical and provides convenient settings for implementing the WHC. However, prior to introducing the WHC into these settings, it is essential to determine whether it is feasible to implement this program in these settings given that they often are under resourced and may not be able to respond to barriers and access to public health services [29, 30]. Identifying potential barriers and facilitators is key to assessing appropriateness, and necessary for adoption [31]. Additionally, understanding the context of the implementation settings may also enhance the feasibility and acceptability of the intervention, which may result in the sustainability of its adoption [31, 32]. Consequently, identifying barriers, facilitators, and any adaptations is a critical first step in the implementation science process [33].

This study presents a first step toward examining the acceptability and feasibility of implementing the WHC in PHC clinics and substance abuse rehab settings in Cape Town, South Africa. Specifically, it explores perceived barriers and facilitators from service providers, key informants, and women on acceptability and how the intervention will need to be adapted to facilitate implementation.

## Methods

### Design

This paper presents the formative, qualitative phase of an implementation hybrid type II study [34]. We conducted focus group discussions (FGDs) with women who use substances and with service providers, we also conducted in-depth interviews (IDIs) with health service planners at local and provincial levels. Our goal was to examine implementation and clinical outcomes associated with delivery of the WHC across PHC clinics and substance abuse rehab programs. This study utilized FGDs to capitalize on within-group dynamics with the women and providers. FGDs with government officials and health service planners was not feasible therefore IDIs were conducted for these participants.

### Participant recruitment and setting

In this initial formative stage, we only conducted two FGDs with women who use substances (*n* = 23), two FGDs with substance abuse rehab providers (*n* = 15), and two FGDs with PHC providers (*n* = 22). See Table 2 for detailed characteristics of FGD participants.

**Table 2:** Focus group discussion (FGD) and individual-expert interview (IDI) participant demographic characteristics

Demographic characteristic	Women Participants FGD	Primary Healthcare Staff FGD	Substance Abuse Rehab Staff FGD	Government Officials/Health Service Planner IDIs
Participants	23	22	15	15
Gender	23 Female 0 Male	21 Female 1 Male	14 Female 1 Male	11 Female 4 Male
Ethnicity	22 Coloured 1 Black African	14 Coloured 6 Black African 1 White 1 Not Reported	8 Coloured 3 Black African 3 White 1 Asian	7 Coloured 1 Black African 7 White
Age Range	19−34	27−51	23−51	Not collected
Description	Women living with or vulnerable to acquiring HIV who use substances	Senior workers, nurse practitioners, clinical managers, counselors, nurse assistants, adherence counselors, senior therapists	Social workers, therapists, treatment coordinators, counselors, program managers, interns	City of Cape Town mayoral committee members, provincial department of health HIV directorates, substance use services managers and treatment directors, department heads, project managers, treatment supervisors, chief directors of health, women’s health managers, provincial ministers

Women were recruited using established street outreach methods proven successful in previous studies for recruiting vulnerable women in Cape Town [35, 36]. Project staff marketed the study in PHC clinics, substance abuse rehab programs, and locations in surrounding disadvantaged communities served by these facilities where potentially eligible women frequent. To be eligible, a woman had to be between the ages of 18 and 35, be Black African or Coloured (mixed ethnicity), have used AODs at least weekly during the past 60 days, had sex with a male partner in the past 60 days, provide contact information, and be willing to talk in a group of peers.

Project staff met with healthcare providers, explained the study, and recruited them to participate in FGDs. To be eligible, healthcare providers had to be working at a PHC clinic or substance abuse rehab program located within a disadvantaged community and have ongoing interaction with patients who met eligibility criteria for the WHC.

We conducted IDIs with key informant health service planners at local and provincial governmental levels (*n* = 15). A list of relevant people in health and social welfare departments was generated based on recommendations from our Community Collaborative Board (CCB) and knowledge of experts in the area (see Table 2).

### Data collection

All FGDs were held in private rooms at PHC clinics and in the substance rehab programs. Prior to the start of each FGD, participants provided written informed consent. A focus group guide consisting of open-ended questions with probes was used to clarify points, encourage continuous dialogue, and guide discussion regarding barriers and facilitators. Participants were briefly shown the cue-card-driven WHC intervention. This paper focuses on the FGD topics related to issues of implementing the WHC in the clinic setting from the perspectives of the women and providers. The US-based Principal Investigator and a South African consultant, both psychologists experienced in group dynamics, facilitated the FGDs.

IDIs with health service planners took place in the participants’ offices. Participants provided written informed consent. Interview guides similar to those used in the FGDs guided the interviews. These interview guides also included questions about the availability of services for the target population, and the extent to which HIV services were integrated with substance abuse rehab services. Participants were also shown the WHC intervention.

FGDs and IDIs lasted between 60 and 90 min each, and were audio-recorded and transcribed verbatim.

### Data analysis

We analyzed the data using a modified grounded theory [37] approach informed by Proctor and colleagues’ conceptualization of implementation outcomes [31]. Two project staff members conducted analyses. First, they created an initial code list based on a preliminary reading of randomly selected transcripts from each participant group. Codes were reviewed and modified during regular meetings between the two project staff members during the process. There were few disagreements regarding the codes. Where disagreements occurred, they were resolved with discussion. Once the two coders had identified a list of primary and secondary codes, they shared the codes and text examples from various transcripts for context with the other members of the research team who further refined the codes. The final coding scheme consisted of four main code families: perceived barriers and facilitators to implementing the WHC; patient barriers; clinical or provider barriers; and operational (or structural) barriers to women seeking treatment or clinicians providing services in general. The current manuscript describes the first code family, perceived barriers and facilitators to implementing the WHC.

We then used a unit of analysis approach [38] to assess intercoder reliability, which was measured using Kappa. Specifically, both team members coded portions of randomly selected transcripts from each stakeholder group, compared the results, and discussed any disagreements in an effort to reconcile them to reach a final coding decision [38]. To prevent agreement persuasion, one coder shared a blinded unit of analysis transcript with the codes removed, though the relevant lines of text remained highlighted for the other coder to code. Through this process, some codes, including one primary code, were merged to clarify coding definitions, whereas new codes were added to properly code emerging themes. Prior to merging the fifth primary code, Kappa for intercoder agreement for the primary codes alone was .91. When intercoder agreement was recalculated to account for the merging of the primary code, Kappa was .90.

## Results

### Participant characteristics

Table 2 presents the characteristics of participants in the FGDs and IDIs. The study included a total of 75 participants that ranged in age from 19 to 51 years.

### Themes

Prior to reviewing the WHC intervention, service providers described the complex, intersecting challenges related to why primary health and substance abuse rehab services are not reaching substance-using women who are HIV positive. Providers revealed intertwined barriers such as IPV; women’s lack of self-sufficiency; drug use; fear and stigma; gang involvement; lack of empowerment; denial of illness; food and income insecurity; lack of steady employment and child care; and operational barriers, including a lack of staffing and poor outreach and follow-up procedures.

#### Barriers to implementing the WHC

Across the FGDs and interviews, themes relating to barriers and facilitators to implementing the WHC in clinics and substance abuse rehab programs emerged. After reviewing the WHC, participants identified concerns with the appropriateness of the program that might interfere with its implementation. These concerns included sensitivity of intervention topics, length of the intervention, delivery format of the intervention, and staffing constraints.

#### Barrier: Sensitivity of intervention topics

The sensitivity of the topics covered by the WHC—such as sexy safer sex and sex communication—was identified as a potential barrier for intervention adoption. Providers and patients expressed being uncomfortable discussing personal and sensitive sexual health topics, and they may feel embarrassed or ashamed. In discussing sexual communication and pleasing their partner, one clinic staff stated:

“They too shy to touch the boyfriend or husband because they say in their culture they doesn’t do that.” [Female, Coloured: PHC Staff FGD1]

The topics may also bring up memories of traumatic experiences for patients or trigger the realization of past abuse. Participants also were concerned that additional services and care that may be needed following the WHC, such as counseling for overcoming previous trauma, are not included as part of the program or available in the immediate health services clinic. Health service planners said:

“The one thing that stuck out for me and that was the rape topic…you’ve now explained to them what rape is, are every one of them going to come and say actually I was raped” [Female, Coloured: Expert Interview]

“You bringing people in, you getting them to the stage where they facing their issues, and they ready to make a change…and then it’s finish and you know that’s …that’s bad and you don’t want to do that.” [Male, White: Expert Interview]

#### Barrier: Length of the intervention

Some participants expressed concern about the WHC being too brief as a two-session intervention. Among some government officials, for example, the short length of the program raised concern about its impact, coupled with the lack of follow-on services available after completion of the WHC sessions. However, other participants were concerned that the intervention would be too long to routinize into ongoing clinical care, considering that providers are already overburdened. Others stated that the time required by staff to commit to training on the program and conducting sessions is too much of an added burden to already overloaded community care workers (CCW). These concerns are reflected in the following examples:

“I think it’s a bit long for…the healthcare workers to do routinely….” [Female, White: Expert Interview]

“I’m generally skeptical of once-off or very short-term interventions having a profound and sustained impact on behavior.” [Male, White: Expert Interview]

#### Barrier: Delivery format of the intervention

Service providers and clinic staff shared their reluctance to conducting the intervention with women on a one-to-one basis or without the male partner’s participation. Staff thought that women would not attend alone because they may fear IPV and self-stigma. However, they did think that the intervention would work better in a group format. Many participants expressed the need to involve male partners in order for behavior change to occur among these women, because women cannot completely change their risk behaviors without their partners also changing their behaviors. PHC staff note that:

“In a group it will work, not one on one.” [Female, Coloured: PHC Staff FGD1]

“… a program like that will only benefit this community if the men would be able to sit in on this program when it’s presented, so that they will also understand the benefits of this, this is not just a women’s thing.” [Female, Coloured: PHC Staff FGD1]

#### Barrier: Staffing constraints

Staffing constraints represented the greatest potential barrier to implementing the WHC. Clinics and substance abuse rehab programs are acutely understaffed and overburdened with a growing number of clinic activities. Additionally, staff often do not have the necessary training to conduct behavioral interventions and to properly communicate with patients. Further, a lack of integration of HIV and substance abuse rehab services makes it difficult to deliver an intervention program such as the WHC because of a knowledge gap in how to deliver collaborative and integrated services. This type of siloed care is often amplified by differential policies and separately allocated government funding for substance abuse rehab and for HIV services. Experts shared their concerns about further burdening untrained clinic staff:

“…people are loading more and more responsibility on these community care workers, which I find a concern.” [Male, White: Expert Interview]

“You know there is not that many people with those skills…. So you got a lot of people that are fairly low to moderate level of skills, and trying to get more sophisticated programs going with people at that skill level can be very difficult.” [Male, Coloured: Expert Interview]

Clinic staff affirmed this concern:

“I think the question is …would we like to adapt to implementing this thing?” [Female, Coloured: PHC Staff FGD2]

“I think the biggest concern is, will we have time to do it.” [Female, Coloured: PHC Staff FGD2]

Some participants shared that even staff who are qualified to conduct such services lack a person-centered approach to interacting with patients. Consequently, treatment by clinic staff raises another potential barrier to reaching patients:

“I don’t remember being trained on how to speak; in fact, what I saw around me from my seniors is almost speaking down to patients and not speaking to them in a person-centered way.” [Female, Black African: Expert Interview]

Referring the women to outside services for additional counseling or support may also be difficult because of limited resources in the community:

“Every time they want to refer somebody to a center there isn’t space there if you are rich then you can, for those that actually need it, most that don’t have the money…there are not enough rehabilitations centers.” [Female, Black African: Expert Interview]

While the issues noted above delineate potential barriers to implementing the WHC across multiple usual care settings, participants also identified potential facilitators, or appropriateness, to implementing the WHC. These facilitators are described below.

#### Facilitators to implementing the WHC

Despite these concerns, three positive facilitators were apparent: high perceived need for the intervention in the community, perceptions that the intervention is empowering to women and establishes personal agency, and perceptions that exposure to the intervention would build staff capacity to better serve substance-using women who are HIV positive.

#### Facilitator: High perceived need for the intervention in the community

Because of the high perceived need for the intervention to address widespread issues in the community, some providers expressed their willingness to implement the intervention despite their concerns about potential barriers. Participants felt the intervention would help to address the intersection of substance use, HIV and violence, including combatting the issue of readily available and easily accessible AODs and limited women-specific services. Consequently, they thought it would be worth investing resources to implement the WHC:

“I think this is a wonderful project and I think we must go all out to try and implement it and test it based on, you know, that we have a high burden of substance abuse and it can be done.” [Female, White: Expert Interview]

“The program is really so nice I wouldn’t like to see you have those kind of troubles because I do believe that you would be able to…if you gonna have a room of ten you going to help at least four or five of those people.” [Female, Coloured: Expert Interview]

“I personally feel strongly that we have to do more for substance abuse and dedicate more resources to it…and sustainability afterwards.” [Female, White: Expert Interview]

Participants in the women’s FGDs also stressed the need for the WHC program, and shared concern around stopping substance use for current and future generations:

“If you open your eyes it’s there (alcohol and other drugs) if you close your eyes it’s there.” [Female, Coloured: Women’s FGD1]

“There is a need [for the WHC], because our children is getting bigger and we don’t want them to see us using that stuff [alcohol and other drugs] and doing that so.” [Female, Coloured: Women’s FGD2]

#### Facilitator: Perceived benefits of empowering women through increased personal agency

In addition to addressing a service gap for substance-using women who are HIV-positive, participants stated that the WHC could positively affect women’s personal agency and decision-making. Government officials, for example, expressed concern around the link of substance use and more complex issues, including a lack of self-empowerment, and felt that the WHC could be beneficial:

“Is that not more to do also with the general sense of hopelessness, a general lack of motivation, a general…lack of self-control and self-empowerment?” [Male, Coloured: Expert Interview]

Clinic staff shared the following regarding the WHC:

“It’s about you know educating, it’s about empowering them because they lack a lot of basic knowledge of self-care.” [Female, Black African: Substance Abuse Rehab Staff FGD3]

“It just feels so nice because they know something about something that they didn’t know and they can self-empower.” [Female, Coloured: PHC Staff FGD1]

Women who were potential recipients of the WHC expressed their excitement about the program and shared that they would attend the program if it was offered at their clinic, and would encourage their friends who also use substances and are at risk to attend as well.

“Everything was useful.” [Female, Coloured: Women’s FGD2]

“We will do this first chance if that happens in our community.” [Female, Coloured: Women’s FGD2]

“We can go at home go say to my friend I learned this, and I learned this today. I would like you to come with us.” [Female, Coloured: Women’s FGD2]

#### Facilitator: Perceived benefits of building staff capacity to better serve women

Clinic and substance abuse rehab program staff and governmental officials recognized the benefits of implementing the WHC in regard to additional training, mentoring, and capacity building for undertrained staff. The project offers training to staff on how to conduct the WHC, how to reach vulnerable women in the community, and how to be sensitive to issues related to sexual health and substance abuse. Participants shared the following:

“I’ve got a nurse’s opinion; okay the nurse’s opinion would be yes this would be good for the community, this would be good in maybe some of us get training….” [Female, Coloured: PHC Staff FGD1]

“In the substance abuse side of things, I think this [is] also sensitizing them, substance abuse service providers around the need for a slightly different emphasis in the approach for woman, I think will be valuable.” [Male, White: Expert Interview]

## Discussion

The WHC is a comprehensive woman-focused approach to HIV prevention and care that addresses the trifecta of AOD use, sexual risk, and IPV. The program has been proven efficacious, but has yet to be implemented in real-world clinical settings. In this study, we explored perceived barriers and facilitators likely to be encountered when implementing the WHC in PHC clinics and substance abuse rehab programs in South Africa.

The main finding is that collectively participants in this formative phase voiced their enthusiasm for an intervention such as the WHC and provided insights into the potential barriers that may need to be addressed for the successful adoption of the WHC. More specifically, there is agreement on the need for such a program, given the risk factors faced by women living with HIV and the lack of effective and accessible substance abuse treatment options available for poor and vulnerable women.

Additionally, all clinic staff, women participants, and stakeholders expressed high acceptability of the WHC. Yet they had concerns about potential barriers to women engaging with this program, largely because of the stigma women may experience when seeking services and the lack of person-centered care at healthcare facilities. Specifically, as previous research has indicated [5, 39], stigma and discrimination are key barriers to women seeking and accessing services for substance abuse and HIV in South Africa.

Another major potential barrier, limited staffing, may impact the ability of clinics to deliver the WHC in addition to their other services. These include the fact that staff members have very little time available to be trained on and to deliver this intervention given their current workload. Additionally, they may not have the requisite skills to deliver this intervention with fidelity.

Finally, participants raised concerns about the length of the intervention and about some of the topics covered in the intervention. This suggests that the intervention content may need to be revised to help insure successful implementation in healthcare settings.

To find potential solutions to these barriers and optimize the implementation of the WHC in healthcare settings, we sought guidance from our CCB, which comprises a diverse group of individuals representing the Department of Social Services and local health departments, alcohol and drug treatment programs, organizations that address victimization and rape, and other community advisors to discuss the progress and challenges of the project. We conducted meetings with CCB members and other key clinic staff to troubleshoot implementation barriers identified during this formative phase of the study.

To address these barriers, the CCB recommended using CCWs rather than nurses to deliver the intervention, mentoring and empowering the CCWs through regular supervision to build capacity to deliver the intervention, and modifying the intervention content to address concerns about intervention length and sensitivity issues raised during the formative phase. Based on the formative findings and feedback from our CCB and clinic representatives, the project team adapted the intervention and modified the implementation procedures to address the participants’ concerns. Specifically, to address sensitivity, the team removed some of the sexually sensitive topics used with previous interventions with sex workers. To address the length of the intervention, some content was reduced, including removing information related to certain drugs that were not relevant to the target clinic populations because typically they are not used in the region. Lastly, to account for staffing constraints in these settings, the study team formed and strengthened partnerships with nongovernmental organizations in Cape Town for on-the-ground support, especially for conducting the intervention.

### Limitations

As with most qualitative studies, the findings may not be generalizable to other settings. However, we have observed similar barriers and facilitators in other settings in the Western Cape and Pretoria. Another limitation inherent in preimplementation formative data collection arises from the fact that participants’ responses and opinions are based on limited exposure to the intervention, as reviewing the intervention is not the same as receiving or delivering the intervention. Nonetheless, our formative data provided critical information that we used to modify the process and the intervention, increasing the likelihood of successful implementation.

## Conclusion

Given that South Africa has the largest percentage of people living with HIV and a disproportionate number of women of childbearing age bearing the greatest burden of the disease, an evidence-based woman-focused intervention that could help women more comprehensively adhere to ART would seem ideal. However, potential barriers exist to implementing the WHC in healthcare settings in resource-scarce environments because addressing clinical crises may be prioritized over the delivery of this woman-focused program. This may be especially true when the program is newly introduced to the healthcare setting, is seen as an add-on to services currently offered by facilities, and is not fully integrated into the usual services delivered by PHC clinics and substance abuse rehab facilities.

The nature of gender inequality across South Africa may evoke mixed feelings among staff about whether the appropriateness of a woman-focused program is needed for the patients they serve. Especially if they hold negative perceptions and stigmatizing opinions of women who use AODs and hold their attitudes as a barrier to engaging with health services. For implementation of the WHC to be successful, women who use AODs must be willing to seek and engage with health services, and providers must also be willing to serve them. To achieve this, we need to reduce health providers’ stigma and discrimination toward women who use AODs, as well as promote empowerment interventions in a country striving for gender equality.

## Abbreviations

AOD: Alcohol or other drugs
ART: Antiretroviral therapy
CCB: Community collaborative board
CCW: Community care workers
FGD: Focus group discussion
IDI: In-depth interview
IPV: Intimate partner violence
PHC: Primary healthcare
WHC: Women’s Health CoOp
